# Deciphering specificity and cross-reactivity in tachykinin NK1 and NK2 receptors

**DOI:** 10.1016/j.jbc.2023.105438

**Published:** 2023-11-07

**Authors:** Jesper J. Madsen, Jacob E. Petersen, Dan P. Christensen, Jakob B. Hansen, Thue W. Schwartz, Thomas M. Frimurer, Ole H. Olsen

**Affiliations:** 1 Global and Planetary Health, College of Public Health, University of South Florida, Tampa, Florida, USA; 2 Center for Global Health and Infectious Diseases Research, College of Public Health, University of South Florida, Tampa, Florida, USA; 3 Department of Molecular Medicine, Morsani College of Medicine, University of South Florida, Tampa, Florida, USA; 4 Section for Metabolic Receptology, Novo Nordisk Foundation Center for Basic Metabolic Research, University of Copenhagen, Copenhagen, Denmark; 5 Embark Biotech ApS, Copenhagen, Denmark

**Keywords:** G protein-coupled receptor (GPCR), mutagenesis, peptide interaction, cross-reactivity, molecular dynamics, molecular modeling

## Abstract

The tachykinin receptors neurokinin 1 (NK1R) and neurokinin 2 (NK2R) are G protein-coupled receptors that bind preferentially to the natural peptide ligands substance P and neurokinin A, respectively, and have been targets for drug development. Despite sharing a common C-terminal sequence of Phe-X-Gly-Leu-Met-NH2 that helps direct biological function, the peptide ligands exhibit some degree of cross-reactivity toward each other’s non-natural receptor. Here, we investigate the detailed structure–activity relationships of the ligand-bound receptor complexes that underlie both potent activation by the natural ligand and cross-reactivity. We find that the specificity and cross-reactivity of the peptide ligands can be explained by the interactions between the amino acids preceding the FxGLM consensus motif of the bound peptide ligand and two regions of the receptor: the β-hairpin of the extracellular loop 2 (ECL2) and a N-terminal segment leading into transmembrane helix 1. Positively charged sidechains of the ECL2 (R177 of NK1R and K180 of NK2R) are seen to play a vital role in the interaction. The N-terminal positions 1 to 3 of the peptide ligand are entirely dispensable. Mutated and chimeric receptor and ligand constructs neatly swap around ligand specificity as expected, validating the structure-activity hypotheses presented. These findings will help in developing improved agonists or antagonists for NK1R and NK2R.

The neuropeptide-dependent signaling system of the neurokinin receptor family (NKR) and their endogenous peptide ligands, tachykinins, have been extensively studied in the past (1). The receptors belong to the G protein-coupled receptor (GPCR) superfamily and consist of three members: NK1R, NK2R, and NK3R, each with its own natural high-affinity peptide ligand (NK1R: substance P [SP], NK2R: neurokinin A [NKA] and NK3R: neurokinin B [NKB]. The three endogenous ligands are highly homologous and share a conserved C-terminal sequence motif known to stimulate some activity towards either of the three NKRs (1). Although the NKRs are attractive drug targets widely spread in cells of diverse tissues, only the effective treatment of chemotherapy-induced nausea and vomiting (CINV) has materialized by NK1R antagonists (1). Still, the intense historical interest in NKR antagonists has indeed resulted in a wealth of highly potent non-peptide compounds for possible drug development. For instance, several selective NK2R antagonists have been considered for the treatment of anxiety disorders (2) or asthma (3); their receptor interaction mode has been discussed based on sequence comparisons (4). Unfortunately, these antagonists have all failed as late as the phase 3 clinical trials. A variety of allosteric modulators have also been explored (5). These and other outcomes call for detailed structural information on NK2R, to serve as a foundation for a better understanding of peptide ligand binding, antagonist function as well as the action of allosteric modulators and their cross-reactivity toward NK1R.

X-ray crystallographic structures of NK1R in complex with a variety of antagonists have recently been reported and form the structural basis of our current understanding of its lack of receptor constitutive activity (among other selective properties) and represent examples of inactive GPCR conformations (6, 7, 8). Furthermore, structures of NK1R in complex with SP and signal mediators, G_q_ and G_s_, have been solved by cryo-electron microscopy (cryo-EM), representing examples of active GPCR conformations of NKR type (9, 10). Recently, a cryo-EM structure of NKA-bound NK2R in complex with the signal mediator G_q_ was resolved (PDB ID: 7xwo) (11). These structural snapshots provide a unique opportunity to explore the conformational changes that form the basis of receptor activation; insofar as the structures represent the ensembles well (12, 13). The binding mode of NKA’s C-terminal to NK2R was found to be identical to that of SP to NK1R (8). However, large differences were found for the N-terminal regions of the two neuropeptides bound to their respective receptor. The advent of ColabFold (14)/AlphaFold2 (15) (AF2) offers accelerated prediction of protein structures and complexes by combining the fast homology search of MMseqs2 (16) with the accuracy of the AF2 deep neural network prediction system (15). Based on AF2 predicted models of NK2R:NKA, NK2R:SP, and NK1R:NKA, these complexes exhibit a truly remarkable capacity for explaining experimental observations from binding, functional, and kinetic assays, which in several key aspects appear to contrast with some of the cryo-EM structures.

We will show that the AF2-predicted models are consistent with the experimental data, both from literature and our own work and is surprisingly effective in explaining the structure–activity relationship of the peptide ligand-bound NKR-type receptors. We identify two favorable interactions on NK2R important for NKA binding, that is, in the β-hairpin of extracellular loop 2 (ECL2) and in the N-terminal region. NK2R-K180 in ECL2 makes a salt bridge with NKA-D4, which in turn forms a hydrogen bond (H-bond) with the mainchain nitrogen of NK2R-A25. The model further predicts that NK2R-F26 forms an aromatic ring interaction with NKA-F6. To further validate the AF2 model of NK2R:NKA:Gq, extensive molecular dynamics (MD) simulations were performed and the key interactions were indeed preserved. In contrast, we find it surprising and unexpected that none of these interactions are identified in the cryo-EM structure. Encouraged by the promising results, we generated models of other complexes to explore cross-reactivity, which remains a significant consideration in the drug development process. As both NK1R and NK2R are of pharmaceutical interest, we focused on their cross-reactivity against NKA and SP. Interestingly, NKA binds to NK1R with fair affinity, whereas SP binds poorly to NK2R. The binding affinity of SP to NK2R is reduced by a factor of 1700 as compared to its binding affinity to NK1R, whereas for NKA, the binding affinity is only reduced by a factor of 37 when it binds to NK1R. By substituting an N-terminal stretch of NK2R this highlights the importance of positions 24 to 26 of NK1R (4). In order to explain these differences in cross-reactivity, models of the complexes NK1R:NKA and NK2R:SP were built. The importance of the β-hairpin of ECL2, as well as differences in sidechains of the N-terminal region 24 to 26 interacting with the N-terminal of the ligands, are substantiated based on the structural models as well as mutated or chimeric receptor, and ligand constructs.

## Results and discussion

### Structural discrepancies between predicted and experimentally resolved NK2R:NKA complex structures

**Figure 1:**
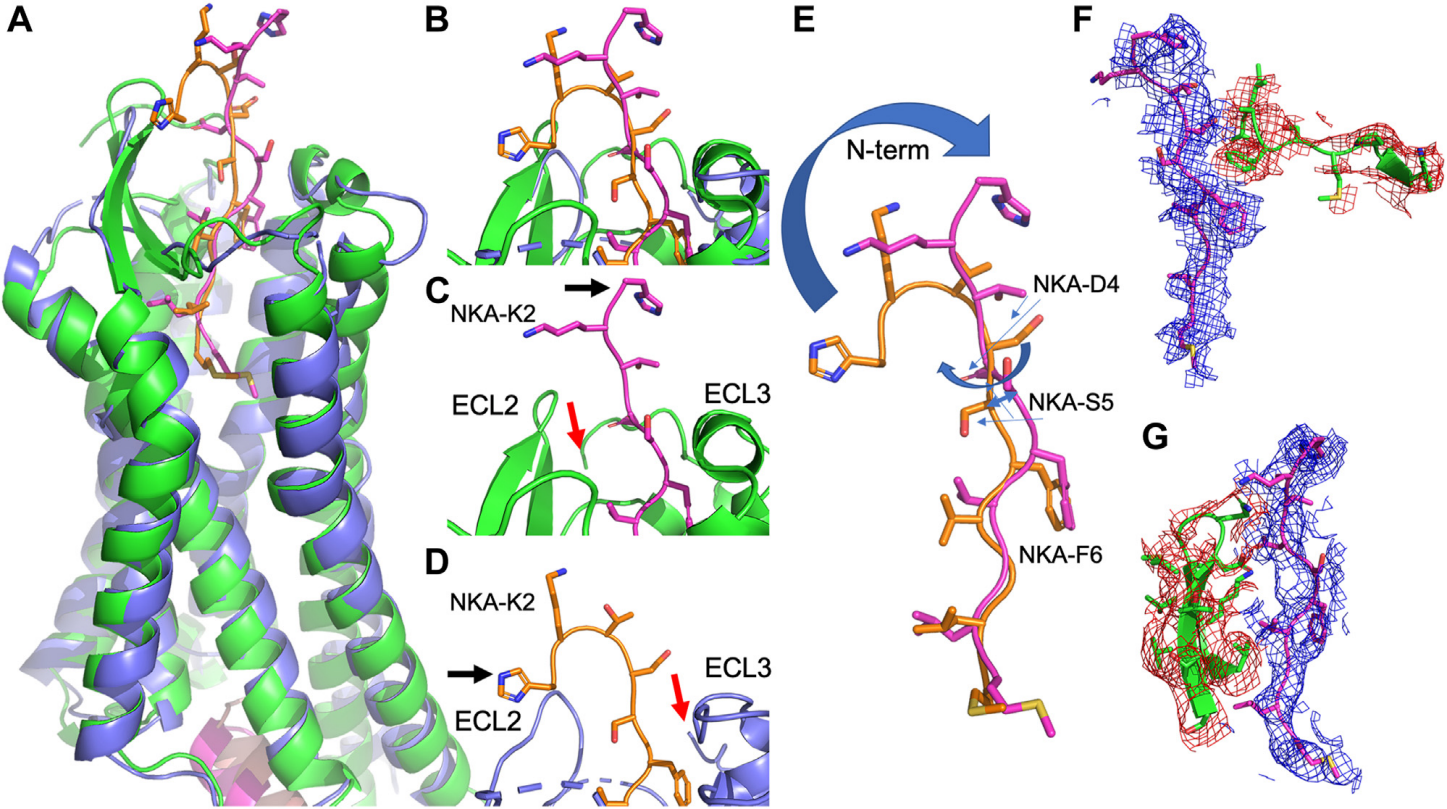
**Comparisons of cryo-EM and AF2 structures of NK2R:NKA.***A*, GPCRs are shown in *blue and green* for the cryo-EM and AF2 structures, respectively. NKAs are positioned in the orthosteric pocket - *brown* in cryo-EM structure and *purple* in AF2 structure. At the intracellular region parts of the G protein C-terminal helices are *brown and purple* for cryo-EM and AF2. *B*, a close-up of the extracellular GPCR regions in complex with their respective NKAs. *C* and *D*, *red and black arrows* point to the N-terminals of GPCRs (S27 for cryo-EM and T21 for AF2) and NKAs (NKA-H1). The positions of the extracellular loops (ECLs) 2 and 3 are indicated. The N-terminal of NKA in the cryo-EM structure interacts with ECL2 while the first 3 residues of NKA in the AF2 structure are solvent exposed. *E*, the overlaid NKAs. The C-terminal regions (NKA6-10) match, while the N-terminal conformations differ. The sidechains of NKA-S5 and NKA-D4 point in different directions as depicted by *blue*, thin arrows. NKA’s first three N-terminal residues are completely solvent exposed. *F* and *G*, comparisons between AF2 model and density maps. In f the interaction between NKA (*purple, blue mesh*) and NK2R N-terminal (*green, brown mesh*) is depicted. In f the interaction between NKA (*purple, blue mesh*) and NK2R ECL2 (*green, brown mesh*) is depicted.

The interaction between NKA and NK2R contains several distinctive structural features, but comparing these complex structures determined by cryo-EM and AF2 highlights important discrepancies among them (Fig. 1). In Figure 1*A*, the transmembrane (TM) part, the orthosteric pocket, and a small part of the N-terminal helix of the G protein are displayed, and a RMSD of 1.3 Å (Cα of TMs only) indicate reasonable overall structural agreement. Comparisons of the N-terminal and ECL2 regions are shown in Figure 1, *B*–*D*. The cryo-EM complex is missing a significant portion of the NK2R N-terminal, beginning only from position NK2R-S27 (Fig. 1*D*). However, experimental results (from the present study) show that the mainchain atoms of NK2R-A25 and the sidechain of NK2R-F24 are important for NKA's binding to NK2R. In the AF2 structure, the mainchain nitrogen of NK2R-A25 forms an H-bond with the sidechain of NKA-D4, while the sidechain of NK2R-F26 forms an aromatic-aromatic interaction with NKA-F6. These are absent in the cryo-EM structure. The cryo-EM structure fails to correctly account for the canonical disulfide bridge between ECL2 and the extracellular end of TM5 (6, 7, 8, 9), as the distances between CB and SD atoms of the cysteines NK2R-C106/C181 are too far apart (7 Å and 4.9 Å, respectively). This disulfide bridge is correctly built in the AF2 structure, leading to entirely different conformations of ECL2 in the two structures (Fig. 1, *C* and *D*). Figure 1*E* shows that the N-terminal conformations of the two NKA structures differ, while their C-terminals are in agreement. It is noteworthy that AF2 predicts the formation of a disulfide bridge between the N-terminal NK2R-4C and NK2R-281C of ECL3 in all predicted structures, capping the receptor in a similar fashion to what is seen in other GPCRs (17). The resulting long protein stretch (∼16 amino acids) is disordered and not predicted to interact with the peptide agonists. Finally, Figure 1, *F* and *G* illustrates a comparison between the AF2 model (NKA, NK2R N-terminal, and NK2R ECL2) and the cryo-EM density data (PDB ID: 7xwo) (11), which in our opinion reveals a convincing match despite abovementioned discrepancies between the cryo-EM and AF2 structures.

**Figure 2:**
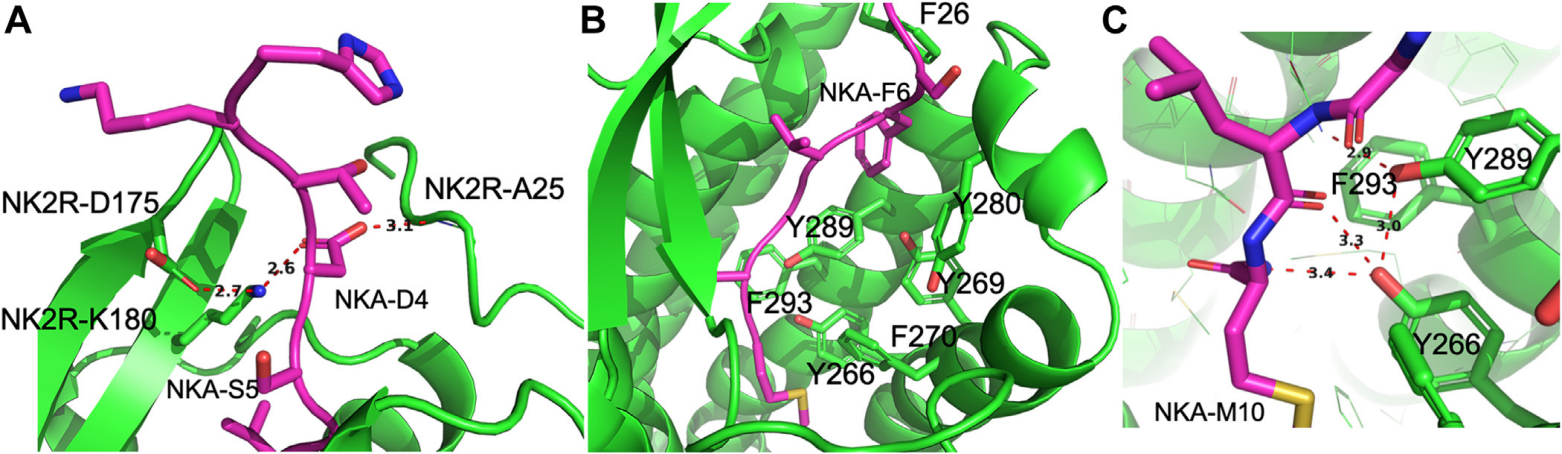
**Importance of NK2R-K180 and NK2R-A25/F26 for NKA binding.***A*, NKA-D4 participates in H-bond/ionic interaction with NK2R: Salt bridge to NK2R-K180 and H-bond to mainchain Nitrogen of NK2R-A25. The orientation of NK2R-K180 is further stabilized by interaction with NK2R-D175. Interactions are depicted by *red dotted lines*. *B*, the involvement of NKA-F6 in the tight network of aromatic rings in the orthosteric pocket is shown. NKA-F6 is sandwiched in between NK2R-F26/Y289. *C*, H-bond pattern between C-terminal of NKA-M10 and NK2R-Y266 not present in NK1R is shown.

Experimental data presented below suggest that the sidechain of NK2R-K180 in ECL2 is important for the binding of NKA. In the AF2 structure, this sidechain forms a salt bridge to NKA-D4 (Fig. 2*A*), and its orientation is further stabilized by an ionic interaction with NK2R-D175. However, NK2R-K180, which is unique for NK2R (Val and Leu appear in NK1R and NK3R, respectively), remains unresolved in the cryo-EM structure even though the cryo-EM density data support the presence of the salt bridge. The H-bond pattern in the NK2R:NKA AF2 model is elucidated in receptor-ligand (“LigPlot+” (18)) diagrams presented in Fig. S1*A*.

Additionally, NKA-F6 is conserved among tachykinins (1) and the residue sidechain forms a tight network of interacting aromatic rings with NK2R-F26 and NK2R-Y289, as shown in Figure 2*B*. However, the interaction between NKA-F6 and NK2R-F26 is not discernable in the cryo-EM structure due to the missing N-terminal segment.

#### NKA residues 1 to 3 are dispensable for the activation of NK2R by NKA

**Figure 3:**
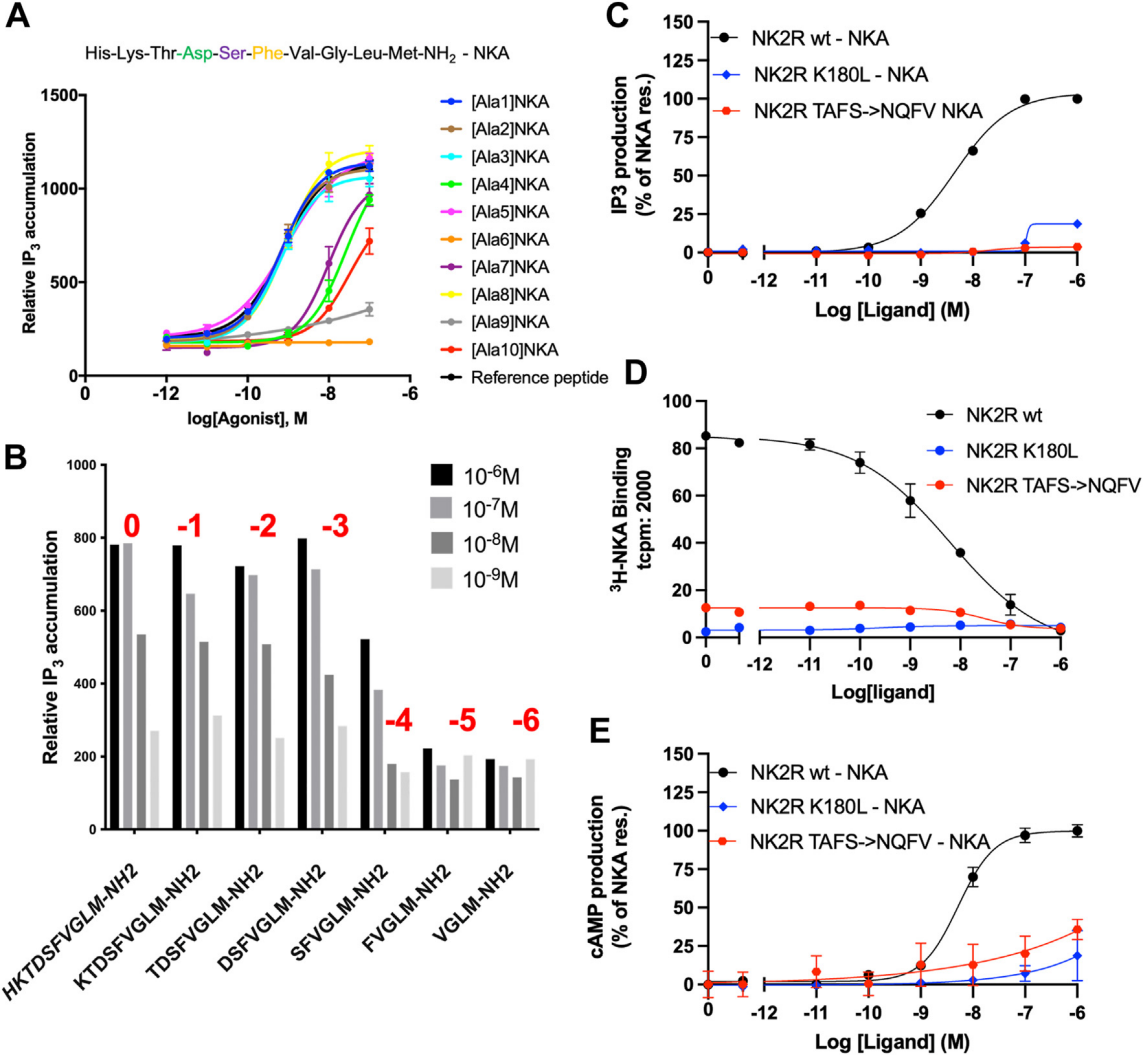
**Experimental data in support of the cryo-EM model**. *A*, the results of an alanine-scan performed on NKA targeting NK2R and tested in dose response in an IP3 assay evaluated in cells. The sidechains of the first three and the fifth amino acid in NKA have no effect on activation in sharp contrast to the effect of alanines in positions 4 (Asp, highlighted in *green*), 6 (Phe, highlighted in *yellow*) as well as the last four positions of NKA. *B*, results from truncated NKA analogs tested in an IP3 assay at four concentrations. The *red* highlights above the columns indicate the number of truncations performed. Activities are preserved for truncations NKA 1 to 3 while truncation of the first 4 amino residues (and more) of NKA results in drastic reductions in activity. *C*, NK2R mutations NK2R-K180L and NK2R-TAFS24 to 27/NQFV are tested in an IP3 activation assay. A dramatic impact on activation is observed in both cases, consistent with the AF2 model. *D*, results from the binding assay showing negligible binding of NKA for the NK2R-K180L and a strongly reduced binding for NK2R-TAFS24 to 27/NQFV. *E*, BRET-based cAMP assay displays remarkable loss of activity for NK2R-K180L. In Table S2 the EC50 and Emax values from functional assays corresponding to panels *A* and *C*–*E* are tabulated.

A truncated version of NKA called GR64349 (KDSFVGL(γ-lactam)M-NH_2_) is a well-known full agonist and the most selective NK2R agonist reported so far (19). An alanine scanning exploration of NKA reveals that NKA-H1A,-K2A,-T3A and -S5A exhibit activity comparable to that of natural NKA in an IP_3_ assay (Fig. 3*A*). In sharp contrast, NKA-D4 and NKA6-10 are found to be critical for activity. The AF2 model shows that the N-terminal of NKA has no interaction with NK2R (Fig. 1*B*), which is consistent with the alanine scanning data. Furthermore, when shortened analogs of NKA were tested in an activation assay, activity was maintained for truncations of NKA1-3, whereas truncation of the first 4 (and 5) residues of NKA resulted in a significant reduction in activity (Fig. 3*B*). These findings agree with data obtained from pharmacological assays of alanine monosubstituted analogs of NKA performed on various organs (20).

#### NKA-D4 interacts with NK2R’s ECL2 and an NK2R N-terminal region

The significance of a negatively charged amino acid in the fourth position of NKA was previously examined by substituting it with conservative alternatives in a truncated version of NKA (DSFVGLM-NH_2_) (21, 22). Ala, D-Asp, and Glu substitutions were tested, and only Glu preserved activity. Figure 2*A* shows the sidechains neighboring NKA-D4. ECL2 containing NK2R-D175 and NK2R-K180, as well as the N-terminal amino acid NK2R-A25, are identified as crucial for binding. The H-bond configuration between NKA-D4 and NK2R reinforces the binding of NKA to NK2R. The three sidechains are unique to NK2R among the NKRs. The AF2 structural model presents several predictions that can be verified: the mutation NK2R-K180L was introduced to investigate the significance of the proposed salt bridge to NKA-D6, and the N-terminal motif TAFS in NK2R was swapped for that found in NK1R (*i.e.*, NQFV). The goal of the latter construct was to abolish H-bond formation from NK2R to NKA-D4 by changing the local conformation or creating a steric clash with NKA-D4. Figure 3*C* displays the activity of the mutants in dose-response tested with NKA in an IP_3_ assay and compared to the response of wildtype NK2R. The activity of NK2R-K180L mutant is negligible, while that of the NK2R-24TAFS27/NQFV mutant is significantly reduced. These findings are reinforced by data shown in Figure 3*D*, where the two NK2R mutations experience significantly reduced NKA binding compared to wild-type NK2R. The drastic reduction in IP_3_ activity is also observed in a bioluminescene resonance energy transfer (BRET)-based cAMP assay (Fig. 3*F*). The role of NK2R-D175 has been addressed in the literature, where mutating the Asp to Ala resulted in a 300-fold reduction in activity in a Ca^2+^-mobilization assay (11). These observations convincingly support the binding mode between NKA and NK2R predicted by AF2 (Fig. 2*A*).

#### NKA-S5 is solvent-exposed

According to the structural model, NKA-S5 appears completely exposed to the solvent. This is consistent with previous work (21, 22), which showed that activity is increased when Ser at position 5 is mutated to Lys or Arg in shortened versions of NKA (DSFVGLM-NH2). The AF2 model suggests that this enhanced activity can be attributed to a favorable electrostatic attraction between the newly introduced Lys or Arg and NK2R-D175.

#### NKA-F6 is coordinated in a hydrophobic network of aromatic NK2R sidechains

NKA-F6 is a conserved residue among tachykinins (1), indicating its potential importance for activity and involvement in receptor interactions. According to the AF2 model, it is sandwiched between NK2R-F26 and NK2R-Y289 (Fig. 2*B*). A change in Y289 to Ala results in a 400-fold decrease in activity in a Ca2+ mobilization assay (11). Similar results are observed when the corresponding Tyr in NK1R is mutated to Ala, which reduces binding affinity for SP by four orders of magnitude (23). Moreover, data on NK1R mutations suggest that the substitution of Phe with Ala at the position corresponding to NK2R-F26 leads to more than four orders of magnitude reduction in binding affinity for SP and NKA (24).

#### NKA residues 7 to 10 are required for activation of NK2R by NKA

The last four residues of tachykinins are highly conserved and necessary for both binding and activation (1). This is also evident from the results of alanine scanning mutagenesis (shown in Fig. 3*A*) and receptor activation assays carried out using shortened NKA constructs (shown in Fig. 3*B*). These findings highlight the perfect compatibility between the C-terminal of tachykinins and the bottom of the orthosteric pocket.

### Validation of the AF2 structural model by MD simulations

**Figure 4:**
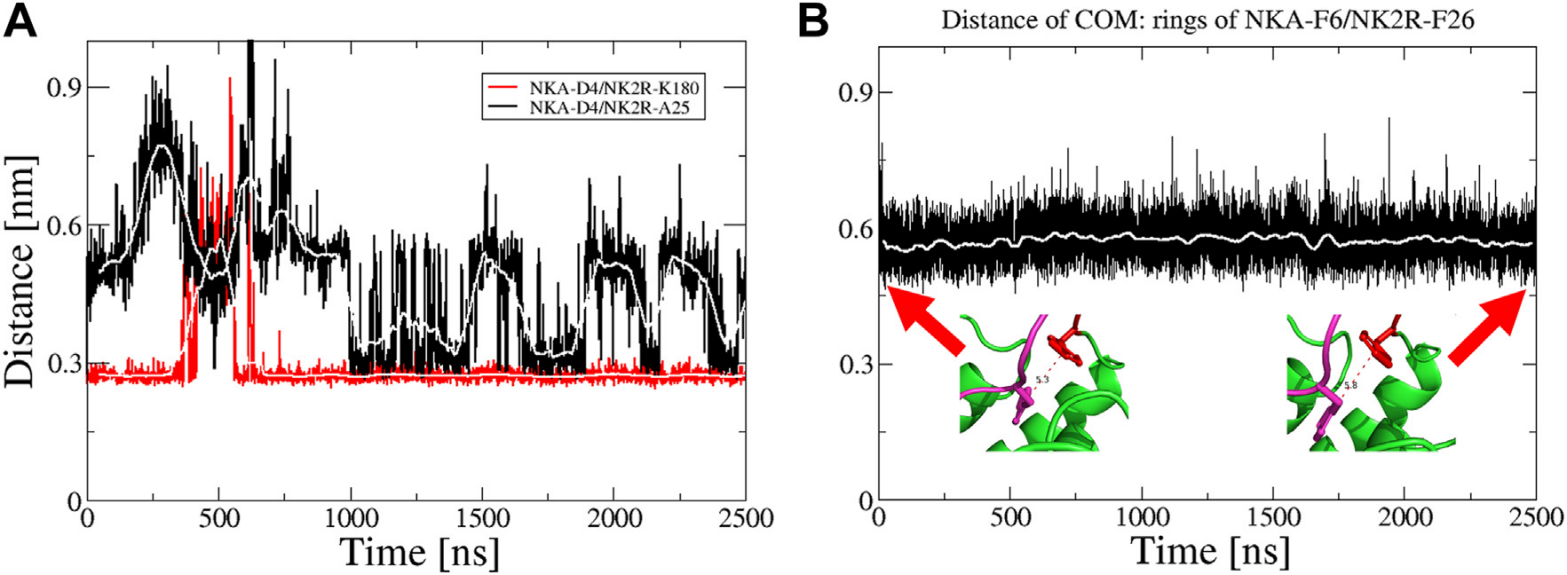
**Results from MD simulations.***A*, ionic interactions: distance (*red*): NKA-D4 carboxylate to NK2R-K180_NZ and H-bond, distance (*black*): NKA-D4 carboxylate to NK2R-A25_NZ. Running averages shown in *white*. The ionic interaction is well preserved, except for a distinct at around t = 500 ns, where mainchain interactions between the N-terminal (NK2R-I23) and the tip of ECL2 (NK2R-A178) breaks the ionic interaction which is restored after another 150 ns. The H-bond is much less stable and settles into a bimodal configuration after about t = 500 ns, where it can be observed ∼50% of the time. *B*, aromatic ring interaction between NK2R-F26 and NKA-F6. Distance between centers-of-mass of the aromatic rings (*black*), running average in *white*. Inserts show the conformations of NKA-F6 (*purple*) and NK2R-F26 (*red*) at T = 100 ns and T = 2450 ns, respectively. The interaction is extremely stable throughout.

The AF2 model was subjected to extensive MD simulations lasting up to 2500 ns to explore and validate it further. The RMS fluctuations for the trajectory were computed for NK2R (Fig. S2) and NKA (Fig. S2, insert). The first three N-terminal residues of NKA experienced significant fluctuations, which is consistent with their high solvent accessibility and contrasted sharply with the buried C-terminal residues. The MD simulation was used to explore the H-bond network responsible for stabilizing the NK2R:NKA interaction (Figs. 2*A* and S1*A*), which involved calculating the distances between the carboxylate of NKA-D4 and the amine of NK2R-K180 as well as the mainchain nitrogen of NK2R-A25 (Fig. 4*A*). The ionic interaction between NKA-D4 and NK2R-K180 was very stable except for a brief interruption at around t = 500 ns when mainchain interactions disrupted it, but it was seen restored after another 150 ns. Conversely, the H-bond NKA-D4/NK2R-A25 was only intermittently observed and adopted a bimodal configuration after t = 500 ns, remaining present about half of the time, likely due to the flexibility of NK2R's N-terminal (Fig. S2). The stability of the aromatic ring interaction between NKA-F6 and NK2R-F26 was assessed by measuring the distance between the center-of-mass (COM) of the aromatic rings (Fig. 4*B*), which was very stable, supporting the experimental observation that this interaction is important. The aromatic rings are placed in energetically favorable stacked conformations (Fig. 4*B*, inserts). The RMS fluctuation of the sidechain atoms of the tight aromatic network, depicted in Figure 2*B*, was computed, revealing that the jump discussed above also occurred at T = 500 ns (Fig. S3, red curve). However, omitting NK2R-F270 from the calculations made the jump disappear, revealing that F270 underwent a conformational change by shifting the rotamer position. This is further elaborated in Fig. S4, where the κ1 and κ2 angles are plotted as a function of time. After the jump, the κ1 angle is predominantly 180 degrees while κ2 shifts between 75 and -120 degrees, indicating a ring flip (Fig. S3, inserts). The change in NK2R-F270 also affected the orientation of the neighboring NK2R-H198. Now, overlay of the cryo-EM structures of NK1R:SP (9) and NK2R:NKA (11) shows that they exhibit same sidechain conformations in this region and that they resemble those illustrated in the left insert of Fig. S3. However, no change in the conformation of NKA-M10.

### Cross-reactivity between NK1R and NK2R and their natural peptide ligands

**Figure 5:**
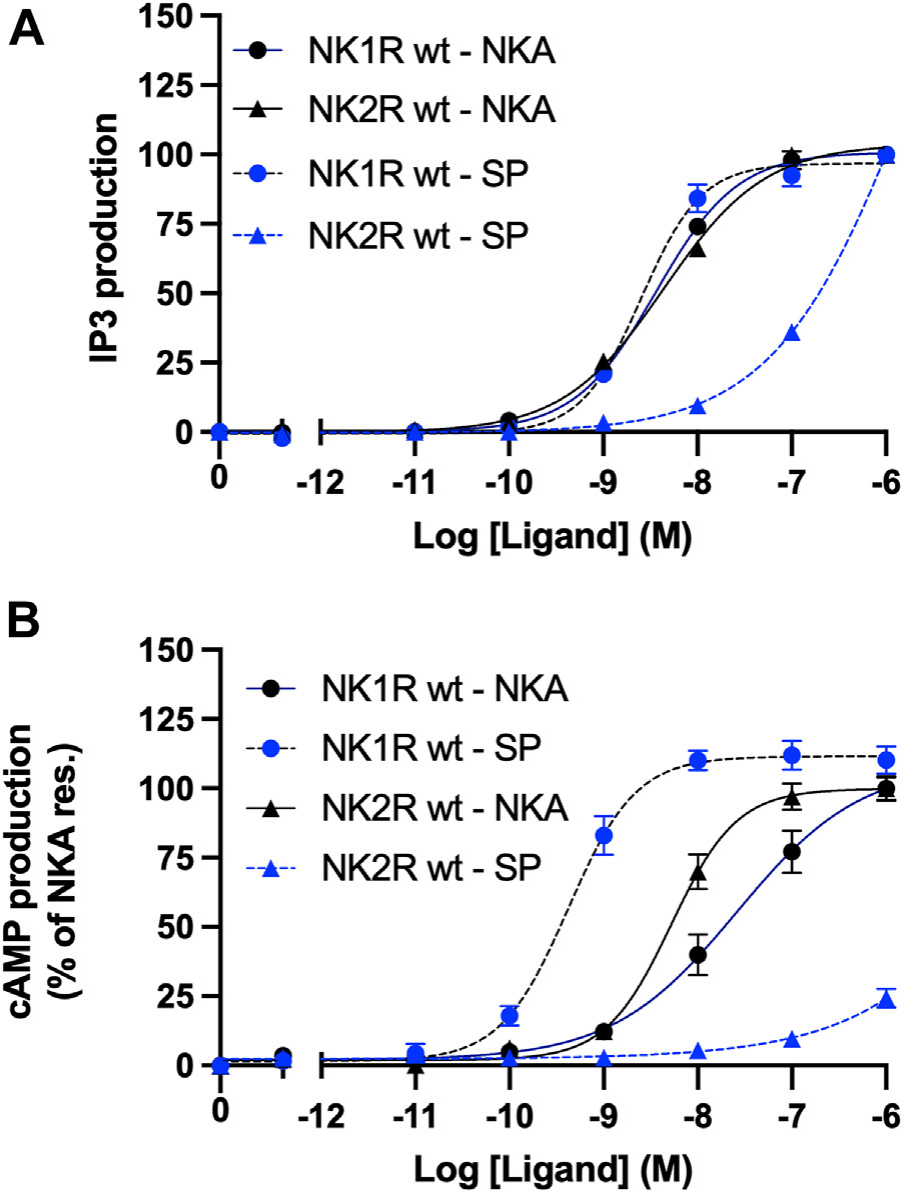
**Activation of NK1R and NK2R.** IP3 (*A*) and BRET-based cAMP (*B*) assays showing cross-activity. SP effectively activates NK1R, it exhibits only weak activation of NK2R. NKA demonstrates the ability to activate both receptors. In Table S2 the EC50 and Emax values from functional assays corresponding to panels *A* and *B* are tabulated.

The remarkable ability of the AF2 model to retrospectively rationalize and predict experimental observations prompted us to model the complexes NK1R:NKA and NK2R:SP. Interestingly, the binding affinity of SP to NK2R is reduced by a factor of 1700 compared to that of NKA. In contrast, NKA binds relatively well to NK1R with a binding affinity reduction of only a factor of 37 compared to that of SP (4). Figure 5 presents additional evidence of the cross-reactivity between NK1R and NK2R by investigating their activation in both an IP3 and a BRET-based cAMP assay. The results highlight that while SP effectively activates NK1R, it exhibits only weak activation of NK2R. In contrast, NKA demonstrates the ability to activate both receptors. This finding suggests that NKA exhibits a broader activation profile compared to SP. It was previously demonstrated by chimeric exchange of transmembrane segments that the binding affinities of SP and NKA for NK1R and NK2R were mainly influenced by changes in the N-terminal regions of these receptors (4). However, the specific sidechain interactions responsible for these differences in binding affinities were unknown at that time. We show that not only are parts of the receptors' N-terminal regions crucial for binding affinity, but also ECL2 regions. We reveal the specific interactions in both ECL2 and the receptor N-terminal regions that explain these differences in binding affinities.

#### Structural comparison of NK1R complexed with SP and NKA

**Figure 6:**
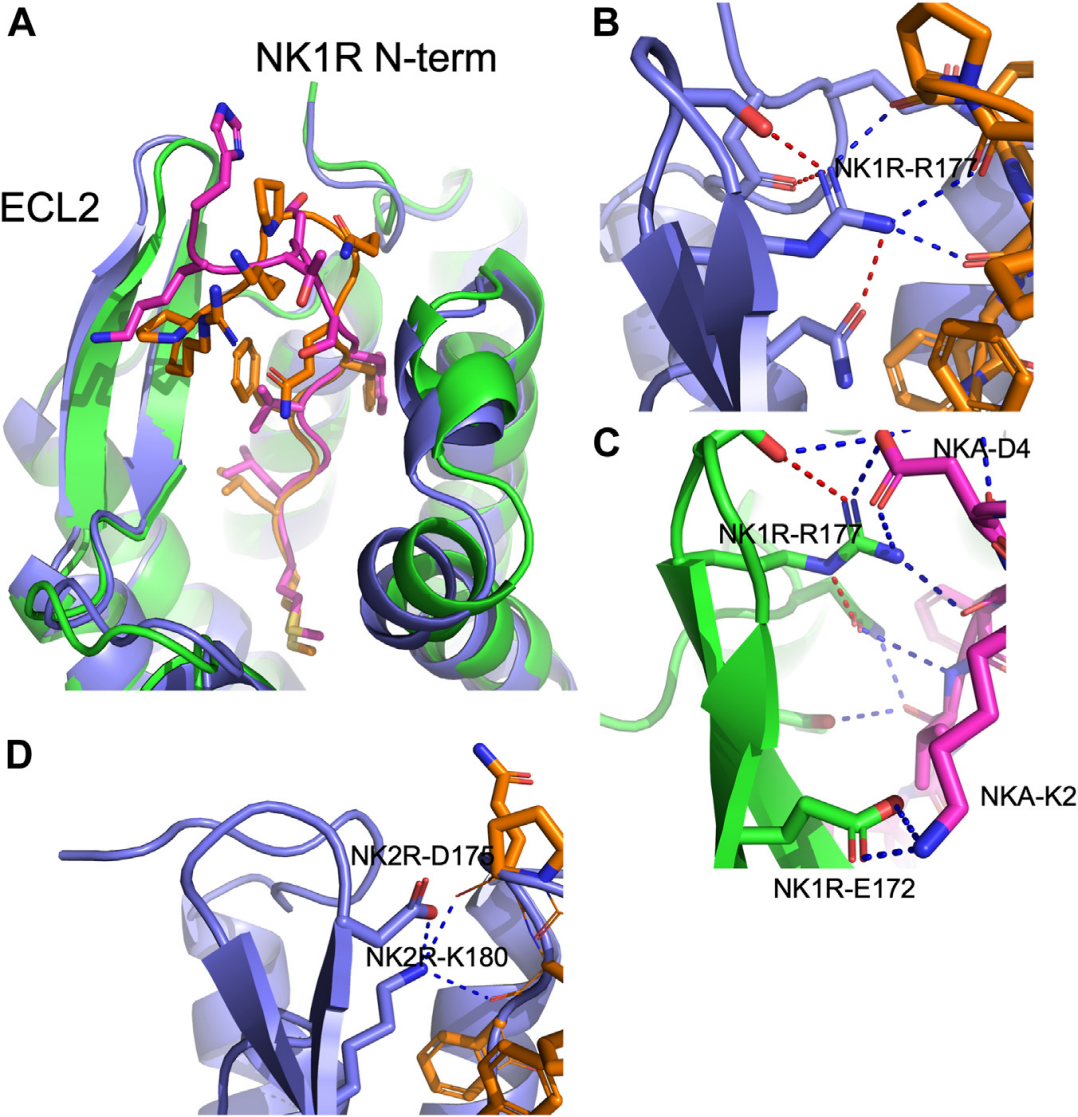
**NK1R:SP cryo-EM structure compared to NK1R:NKA AF2 model illustrating ligand binding modes**. Similar binding of the ligands C-terminals and different interaction of their N-terminals with NK1Rs ECL2 and N-terminal. *A*, GPCRs in cartoon, NK1R(*blue*):SP(*brown*), NK1R(*green*):NKA(*magenta*). The C-terminal 6 amino acids of SP and NKA show perfect overlay, and deviation at their N-terminal starting at residue number 6 (SP) and 5 (NKA). *B* and *C*, H-bonds in NK1R and NK2R in *red*, and H-bonds between GPCRs and ligands in *blue*. In NK1R:SP complex, the strategically placed NK1R-R177 guanidinium group participates in dense H-bond network with sidechain of NK1R-96 (which interacts with SP-F8CO), mainchain CO’s of SP-K3, -P4, and -Q6, and to sidechain of NK1R-S176 (placed at the tip of ECL2) as well as NK1R-N23 sidechain (in NK1R N-terminal). Further, NK1R-Q24 sidechain generates H-bond to SP-Q5-CO. Hence, no sidechains of SP participate in H-bond interactions with NK1R. In NK1R:NKA NK1R-R177 is also placed in a tight H-bond network when binding NKA *i.e.* it interacts with the sidechain of NK1R-96 (which also H-bonds to NKA-V7-CO) and NKA-S5-CO. In contrast to SP a sidechain of NKA, NKA-D4, forms a salt bridge interaction to NK1R-R177 as well as to the sidechain of NK1R-N23 while NK1R-Q24 sidechain generates an H-bond to NKA-D4-CO. The salt bridge between NKA-K2 and NK1R-E172 is depicted at the *bottom* of (*C*). *D*, close-up of the interaction between NK2R and SP in the AF2 model of the complex NK2R:SP. No interactions between SP and the N-terminal of NK2R are predicted. However, the sidechain of NK2R-K180 has the possibility of interacting with the mainchain carbonyls of SP.

Figure 6*A* presents a comparison of the cryo-EM structure of NK1R:SP with the AF2 model of NK1R:NKA. The H-bond networks are described in detail in the figure legend of Figure 6 and visualized in the LigPlot+ diagrams found in Fig. S1*B* (NK1R:SP) and S1c (NK1R:NKA). Both structures exhibit tight H-bond networks between the ligands and NK1R, particularly to the N-terminal region of NK1R. Notably, it has been observed that swapping the NK1R-21 to 29 sequences to that of NK2R in NK1R (making it more like NK2R) leads to a significant decrease in NKAs binding affinity for the NK1R mutant (by a factor of more than 300) (24). The crucial segment of this stretch was identified as NK1R-24NQF since any change in each of the three sidechains to either Thr, Ala or Ala produces similar reductions (24).

**Figure 7:**
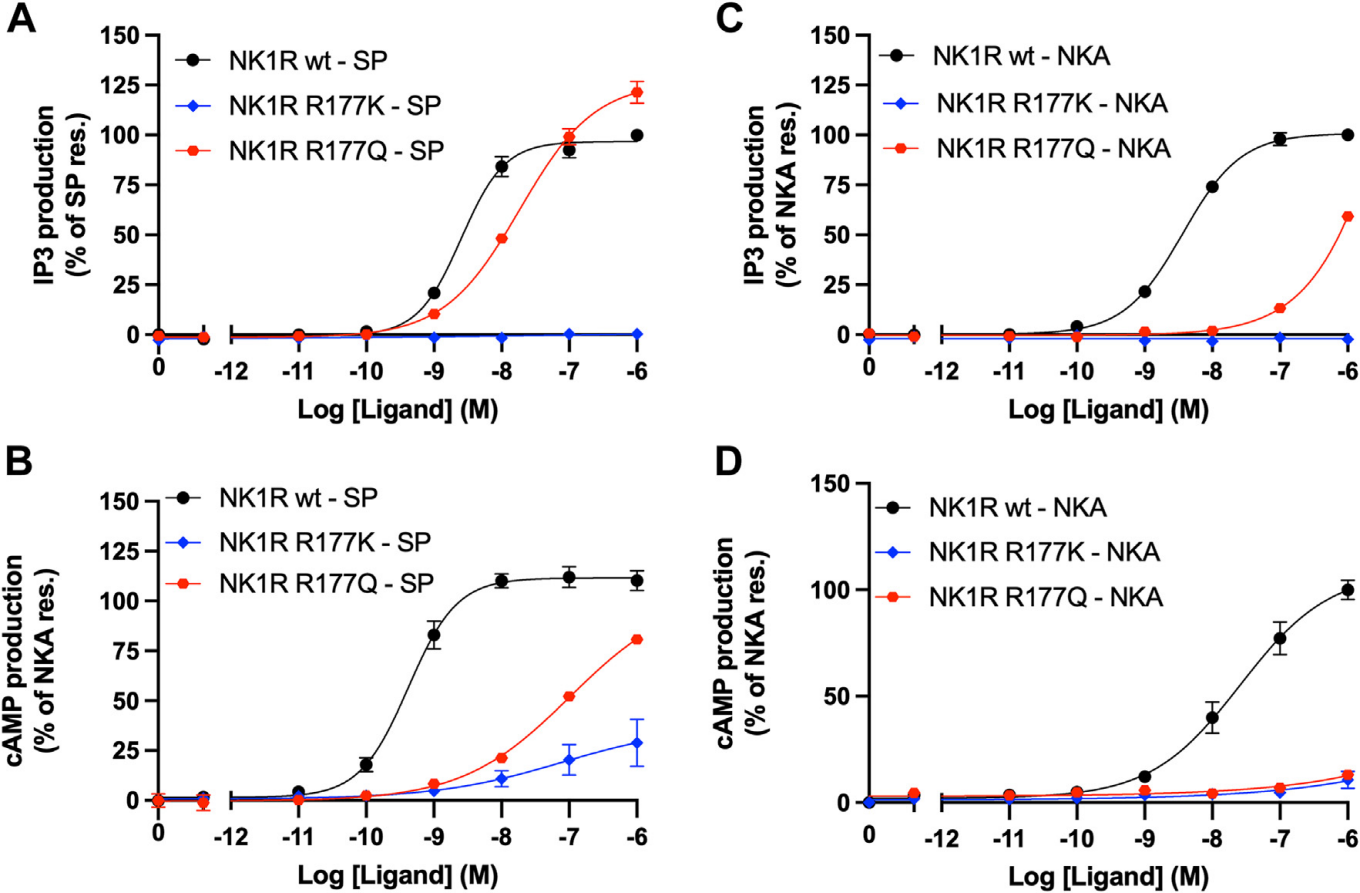
**NK1R mutations NK1R-R177K and NK1R-R177Q.** IP3 activation assay (*A* and *C*) and in BRET-based cAMP assay (*B* and *D*). A dramatic impact on activation is observed in all cases, consistent with the AF2 model. Impact of the Glutamine mutant is somewhat lower than that of Arginine because it can re-establish H-bonds, but not to the extent as observed for Arginine. In Table S2 the EC50 and Emax values from functional assays corresponding to panels (*A*–*D*) are tabulated.

According to the AF2 model of NK1R:NKA, Asn and Gln sidechains in the NQF motif form H-bonds to the mainchain of NKA, linking it to the N-terminal of NK1R. As discussed earlier, NK1R-F27 is also important in this interaction. Hence, mutations in the residue 24 to 26 segment (relative to NK2R) result in a lack of proper interaction between NKA and NK1R. The AF2 model indicates that NKA interaction with NK1R N-terminal is similar to that of SP, except for the significant NKA-D4 sidechain, which forms a salt bridge to NK1R-R177. Figure 6, *B* and *C* depict the interactions observed in the NK1R:SP and NK1R:NKA complexes, respectively, highlighting a potential involvement of the guanidinium group of NK1R-R177 in forming extended H-bond networks. To validate this observation two conservative mutations were introduced, NK1R-R177K/Q. Figure 7 shows the response observed in activation assays performed on the mutants to both SP and NKA. NK1R mutations NK1R-R177K and NK1R-R177Q were tested in IP3 activation assay (Fig. 7, *A* and *C*) and in BRET-based cAMP assay (Fig. 7*B* and 7 days). Interestingly, both mutants drastically influence the activity. However, the change from Arginine to Lysine abolish the activity in both assays supporting the presence of the extended H-bond network. These findings underscore the significance of NK1R-R177 in mediating critical interactions within the ligand-receptor complexes. Therefore, the suggested H-bond networks in the NK1R:NKA model agree with the experimental observation of NKA's activation profile and decent binding affinity to NK1R. In further support of the NK1R:NKA model, mutagenesis data (25) indicate that the NK1R-T170K mutant reduces NKA's binding affinity by a factor of 2.8, while the substitution of E172K reduces its binding affinity by a factor of 12 (Fig. 6*C*) (25). The model further predicts a salt bridge between NKA-K2 and NK1R-E172, which will be broken by electrostatic repulsion in the latter mutation, explaining the lowered affinity. The former mutation may attract NK1R-E172 due to its proximity, thereby weakening its interaction with NKA-K2. Additional support for the role of position NKA-K2 in the complex is the observation that mutation of NK1R-E183 to Gln, which is approximately 5 Å from NKA-K2 in the model's C-terminal end of ECL2, reduces the binding affinity by a factor of 2 (25).

Results from the chimeric exchange of transmembrane segments point to intriguing interplays between ECL2 and the N-terminal regions of NK1R and NK2R (4) (Table S1). A chimeric construct combining an N-terminal part of NK1R and the C-terminal part of NK2R (residue 1–195 including ECL2, NK2R196–398) shows excellent binding of SP (0.14 nM) and reduced binding of NKA (Table S1). However, another chimeric construct including ECL2 of NK2R (*i.e.* NK1R1-130, NK2R131–398) displays a reduced affinity for both receptors (SP binding reduced 36 times, NKA binding reduced 37 times). Binding to NK2R shows a dramatic reduction for SP binding (3300-fold) and the wildtype binding of NKA (0.9 nM). These observations are in support of the structural considerations discussed above.

#### Structural comparison of NK2R complexed with SP and NKA

To account for the reduced binding affinity of SP to NK2R, we also explored the AF2 model of the NK2R:SP complex. The major interactions between NK2R and SP are depicted in Figure 6*D*, along with a corresponding LigPlot diagram in Fig. S1*D*. In the NK1R:SP complex, the two sidechains of NK1R-N24/Q25 link the NK1R N-terminal to ECL2 and SP, respectively. NK1R-N24 forms H-bonds with the NK1R-R177 sidechain and NK1R-H95 mainchain carbonyl, while NK1R-Q25 H-bonds to the mainchain carbonyls of SP-Q5 and NK1R-L179. These sidechain interactions are missing in NK2R, abrogating the contact to NK2R’s N-terminal stretch, and explaining the loss of affinity. As shown in Figure 6*D*, there are no H-bond interactions between SP and the N-terminal of NK2R. However, the model suggests that the sidechain of NK2R-K180 has the potential to interact with mainchain carbonyl groups of SP-P4 and SP-Q6. To substantiate this observation, the two NK2R mutants (NK2R-K180 L and NK2R-24TAFS27/NQFV) discussed above were activated by SP in activation assays (results depicted Fig. S5), resulting in a marked activity reduction for both mutants in the IP_3_ assay. However, in the cAMP assay, the NK2R-24TAFS27/NQFV shows improved activation compared to that of NK2R, consistent with the mutant being more NK1R-like. These observations are supported by the interplay between ECL2 and the sidechain N-terminals of NK1R and NK2R, as described above and summarized in Table S1.

### Perspectives

The results of this study highlight novel avenues for improving the prospects of developing highly specific agonists or antagonists against NK1R and NK2R. First, the remarkable predictive power of AF2 in determining the peptide-ligand-bound receptor structures has been robustly validated and is expected to be widely applicable. The predicted models presented here have demonstrated an exceptional ability to explain experimental observations. Second, we note that the majority of NK1R antagonists (10) exhibits minimal overlap with SP in the receptor orthosteric binding pocket. Therefore, there may be potential for designing superior antagonists by focusing on the receptor binding site of the C-terminal part of SP (7). As such, we propose that the development of high-specificity compounds should target the binding regions corresponding to positions NKA-D4 and SP-Q5. These insights are likely transferrable in principle when designing drugs to target various other GPCRs with endogenous peptide ligands.

## Conclusion

The tachykinin receptors, NK1R and NK2R, are of great pharmaceutical interest. The advent of structures for ligand-bound complexes (9, 10, 11) prompted us to investigate the detailed structure-activity relationship of the ligand-bound receptor complexes that facilitate both high specificity toward the natural ligand but also permit cross-reactivity. First, we applied AF2 to generate models of the NK2R:NKA complex. Comparison of the conformation for bound NKA, the β-hairpin of ECL2 (including the disulfide bridge linking the loop to TM5), and the NK2R N-terminal to the cryo-EM density data shows a perfect match which in several key aspects appear to contrast with the cryo-EM structure. Secondly, this model showed a remarkable capacity to explain experimental observations from binding, functional, and kinetic assays. The model points to the vital importance of K180 and the N-terminal stretch NK2R-24TAF which has been validated by mutagenesis, binding, and kinetic data. The specific interactions of the bound NKA to NK2R have been supported by an alanine scan and functional data of mutated NKA. Hence, the AF2 model explains previously published experimental data as well as experimental data reported in the present work. Thirdly, the cross-reactivity of SP and NK2R as well as NKA and NK1R was addressed. SP binds poorly to NK2R while NKA binds well to NK1R (26). AF2 models of the complexes reveal that specificity and cross-reactivity of the peptide ligands can be completely understood by intricate interactions between the two amino acids prior to the FxGLM consensus motif of the bound peptide ligand and the β-hairpin of ECL2 and N-terminal region leading into TM1. In particular, positively charged side chains of ECL2 play vital roles, that is, R177 of NK1R and K180 of NK2R. The N-terminal positions 1 to 3 of the peptide ligand are dispensable. Mutated and chimeric receptor and ligand constructs neatly swap around ligand specificity as expected, validating the structure-activity hypotheses presented.

The findings of this study suggest promising directions for improving upon specific agonists or antagonists. The study also underscores the effectiveness of AF2 in reliably predicting peptide-receptor interactions for GPCRs, which likely is going to be useful across various applications.

## Experimental procedures

### Structural modeling

AlphaFold2 was used to predict and explore structural models of NK1R:SP, NK1R:NKA, NK2R:SP, and NK2R:NKA complexes. We use the publicly available IPython notebooks to access ColabFold/AlphaFold2 (14, 15, 16). Relaxation of the structures by gradient descent in the Amber99sb force field (27) was done with OpenMM v.7.3.1 (28). Structural visualization was performed in PyMOL (open_source version 2.5.0, Schrödinger LLC, http://www.pymol.org/pymol). Predictions of NK2R:SP required seeding of a template structure (PDB ID: 7p00) to produce a reasonable result.

Structure files deposited: NK1R_NKA.pdb, NK1R_SP.pdb, NK2R_NKA.pdb, and NK2R_SP.pdb in https://doi.org/10.5281/zenodo.8074032 *i.e.*, models discussed in the text.

### Molecular dynamics simulations

Input scripts for the MD simulation of the model of NK2R:NKA (in complex with the helical C-terminal part of G_q_) generated by AF2 were prepared in CHARMM-GUI (29, 30). The standard input generator for membrane builder (bilayer builder) was used. The position of the membrane bilayer was calculated using the PPM server (31). The system was solvated using 22,270 TIP3P waters (32)in a rectangular box (85 × 85 × 135 Å^3^). The ionic strength of 150 mM was obtained by adding Cl^-^ (60) and Na^+^ (243) ions and the membrane was composed of DOPS lipids (192 lipids). The simulations were performed at 303 K using a Nosé-Hoover thermostat in an NPT (constant pressure using Parrinello-Rahman barostat (33)) ensemble using Gromacs (34) and the CHARMM36m force field (35). The system was minimized and simulated for 2500 ns. The trajectory was analyzed using Gromacs tools and in-house scripts. Plotting was done using Grace (xmgrace; https://plasma-gate.weizmann.ac.il/Grace/).

### Compounds

All compounds were dissolved in 100% DMSO or 70% EtOH. NKA and SP were from Sigma Aldrich (N4267 and S6883, respectively). [3H]-NKA was produced by Novo Nordisk A/S for Embark Biotech ApS (Denmark). NKA variants for alanine scan and truncational analyses were produced by Peptides and Elephants GmbH (Germany).

### Plasmids

All receptor constructs of wildtype and modified human *TACR1* and *TACR2* were inserted into the pcDNA3.1(+)-C-DYK vector (Genscript) whereas CAMYEL (36) was expressed *via* pcDNA3.1(+) vector.

### Cell culture, plating, and transfection

COS7 cells (derived from African green monkey kidney fibroblast and originally bought from ThermoFisher) were maintained in Dulbecco´s Modified Eagle´s Medium 1885 with GlutaMAX supplemented with 10% fetal bovine serum (Sigma-Aldrich), 100 units/ml penicillin and 100 μg/ml streptomycin at 37 °C with 10% CO_2_. COS7 cells were plated in either clear-, white-, or white with clear bottom 96-well plates that were coated with poly-D-lysine (Sigma-Aldrich) 30 min prior to cell seeding (20.000 cells/well). The following day plates were transiently transfected using calcium precipitation with 200 ng/well DNA in a culture medium with the addition of 100 μM (final concentration) Chloroquine. Transfection was stopped 5 h later with the addition of a fresh maintenance medium. Fig. S6 illustrates the consistency in expression levels as determined through the Elisa assay for both wildtype receptors and mutants, demonstrating robust reproducibility. The COS7 cells were tested for *mycoplasma* once a month and were negative.

### BRET-based cAMP assay

The intracellular level of cAMP was monitored in real-time using bioluminescence resonance energy transfer (BRET). This was achieved by implementing a construct consisting of a cAMP binding protein (Exchange protein activated by cAMP (Epac)) which has been flanked by a BRET pair consisting of Renilla luciferase (Rluc) and yellow fluorescent protein (YFP). This construct is called CAMYEL (cAMP sensor using YFP-Epac-Rluc) and enables cAMP production to be sensed as Epac changes conformation in response to increasing levels of cAMP, ultimately resulting in a loss of BRET signal.

On the day of the assay, white 96-well plates with COS7 cells were washed twice with 100 μl/well HBSS (GIBCO, Life Technologies) and preincubated for 30 min at 37 °C with 85 μl HBSS. Luciferase substrate coelenterazine h (ThermoFisher) was added and a baseline measurement was taken after 5 min. Dose-response curves of either NKA or SP were added and measurements were recorded every minute for 30 min on a CLARIOstar Plus plate reader. The BRET signal was calculated as the ratio of emission intensity at 535 nm (citrine) to the emission intensity at 475 nm (luciferase). Determinations were made in triplicates.

#### IP accumulation assay

COS7 cells seeded in clear 96-well plates were incubated with 0.5 μCi/ml myo [3H]inositol (PerkinElmer) in 100 μl growth medium over night following the transfection. The subsequent day cells were washed twice with 200 μl/well HBSS (GIBCO, Life Technologies) and pre-incubated for 5 min at 37 °C with 100 μl/well HBSS buffer supplemented with 10 mM LiCl. Ligand addition was followed by 150 min incubation at 37 °C. To stop ligand incubation, cells were lysed with 40 μl 10 mM formic acid followed by 30 min incubation on ice. 35 μl of the lysate was transferred to white 96-well plates together with 60 μl of 1:8 diluted YSi SPA scintillation beads (PerkinElmer). Plates were sealed and vigorously shaken for 15 min followed by 5 min centrifugation at 1500 rpm. Measurements (scintillation) were recorded on a Microbeta (PerkinElmer) after a 4 h delay and determinations were made in duplicates.

#### Competitive binding assay

For the binding COS7 cells were seeded in clear bottom white plates (Costar #3610). Assay solutions were Tachykinin receptor (TKR) buffer: 50 mM Tris/HCl pH 7.5 (GIBCO #15567–027), 5 mM MnCl_2_ (Sigma 63,535) and 150 mM NaCl (Sigma S7653); Wash buffer: TKR buffer + 0.1% BSA (Sigma A7030); Binding buffer: Wash buffer + 0.1 mg/ml Bacitracin (Sigma B0125); Tracer solution: Binding buffer + [3H]-NKA.

Everything was placed on ice. Plates were washed with 200 μl cold wash buffer followed by 100 μl of cold binding buffer. Plates were left on ice to cool down to around 4 °C. A 8-point dilution curve of NKA was added followed immediately by 100 μl cold tracer solution. Plates were left to incubate for 3 h at 4 °C. Binding was stopped by two washes with 100 μl cold washing buffer followed by the addition of 225 μl Ultima GOLD LLT (#6013371) scintillation fluid. Plates were left on medium shaking for 30 min and left O/N at RT. The following day plates were measured on a Microbeta (PerkinElmer) for 10 min/well. Determinations are made in triplicates.

### Statistical analysis

All dose-response curves have been calculated using Prism 10 software (GraphPad Software) non-linear regression with four parameters. All data are shown as mean ± SEM unless stated otherwise and consist of two technical replicates from three biological replicates. In Table S2 the EC50 and Emax values from functional assays are tabulated.

## Data availability

Structure files deposited: NK1R_NKA.pdb, NK1R_SP.pdb, NK2R_NKA.pdb, and NK2R_SP.pdb in https://doi.org/10.5281/zenodo.8074032 *i.e.*, models discussed in the text. All other data are contained within the manuscript.

## Supporting information

This article contains supporting information.

## Conflict of interest

D. P. C. and J. B. H. are employees of Embark Biotech ApS. All other authors declare no competing interests.
