# Supplementary material for: Deciphering specificity and cross-reactivity in tachykinin NK1 and NK2 receptors
Source: J Biol Chem. 2023 Nov 7;299(12):105438. doi: 10.1016/j.jbc.2023.105438 (PMC10724690; doi:10.1016/j.jbc.2023.105438)
Supplement: Supporting information [file mmc1.docx]

**Supporting Information:**

**Tables, Figures and Figure legends**

Structure files deposited: NK1R_NKA.pdb, NK1R_SP.pdb, NK2R_NKA.pdb and NK2R_SP.pdb in <https://doi.org/10.5281/zenodo.8074032>.

**
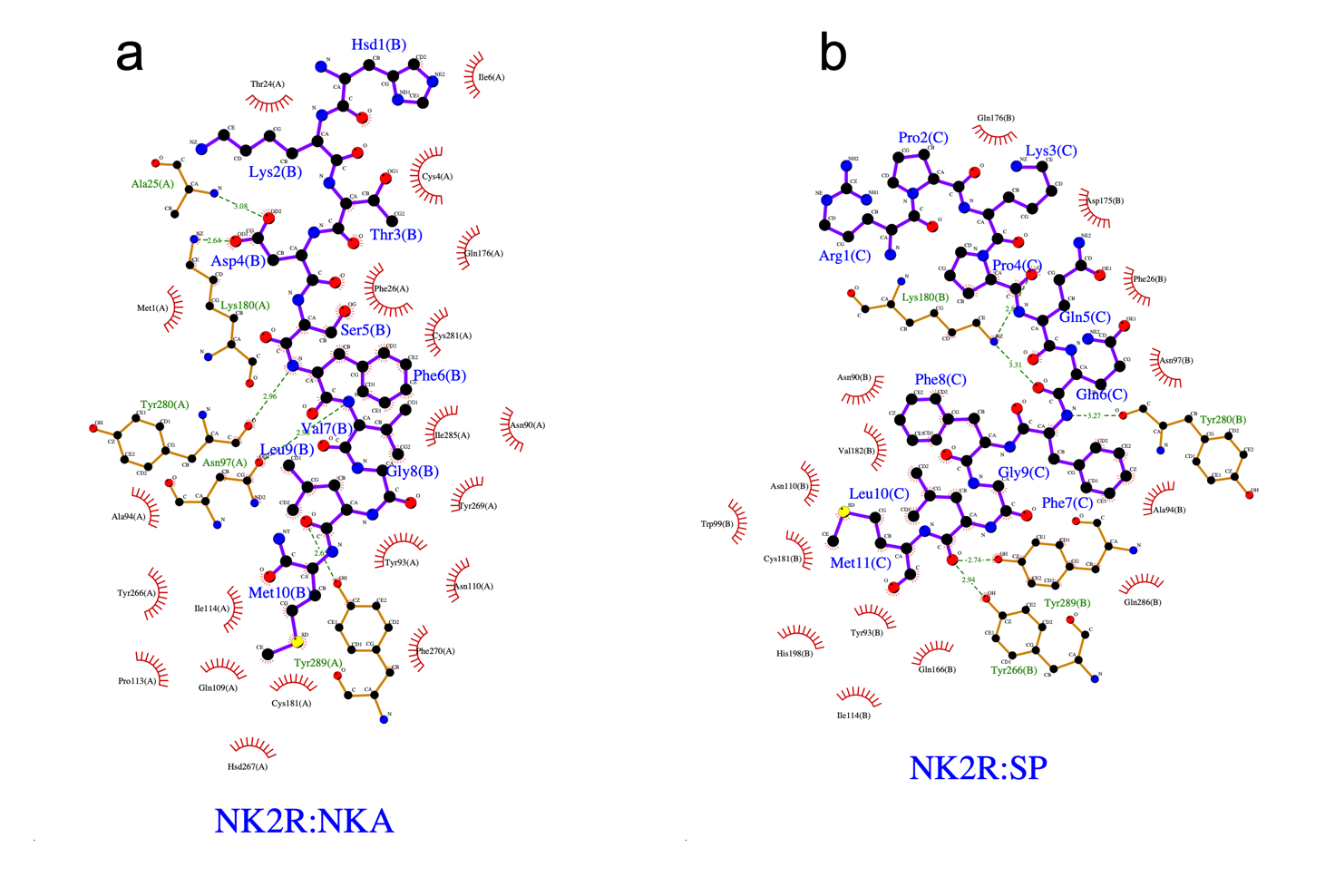
**

**
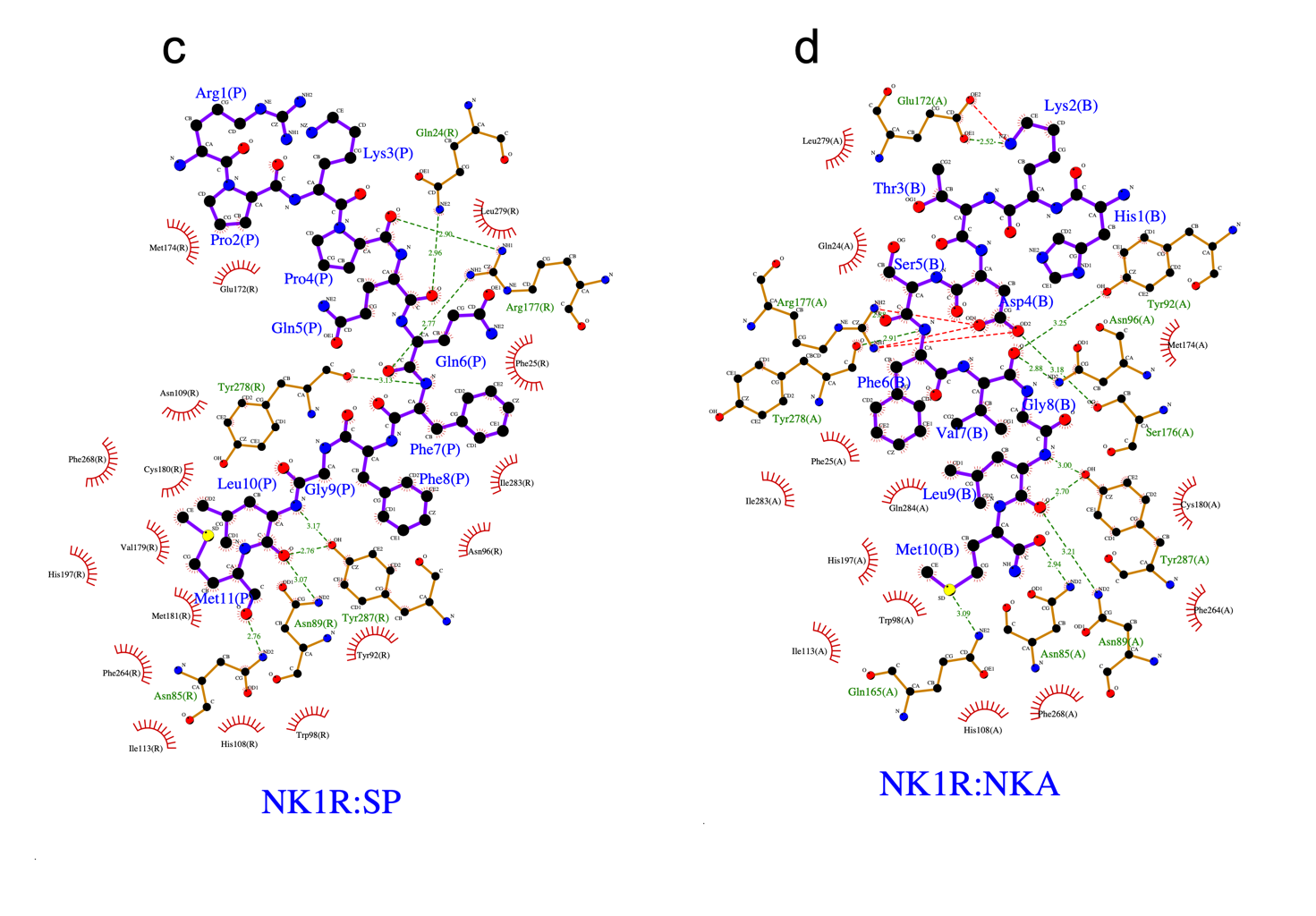
Figure S1:** Ligand binding elucidated in LigPlot+ diagrams of complexes: (a) NK2R:NKA, (b) NK1R:SP, (c) NK1R:SP and (d) NK2R:SP. Peptide ligands are in blue and dark colours. The NKR’s are in light colours.


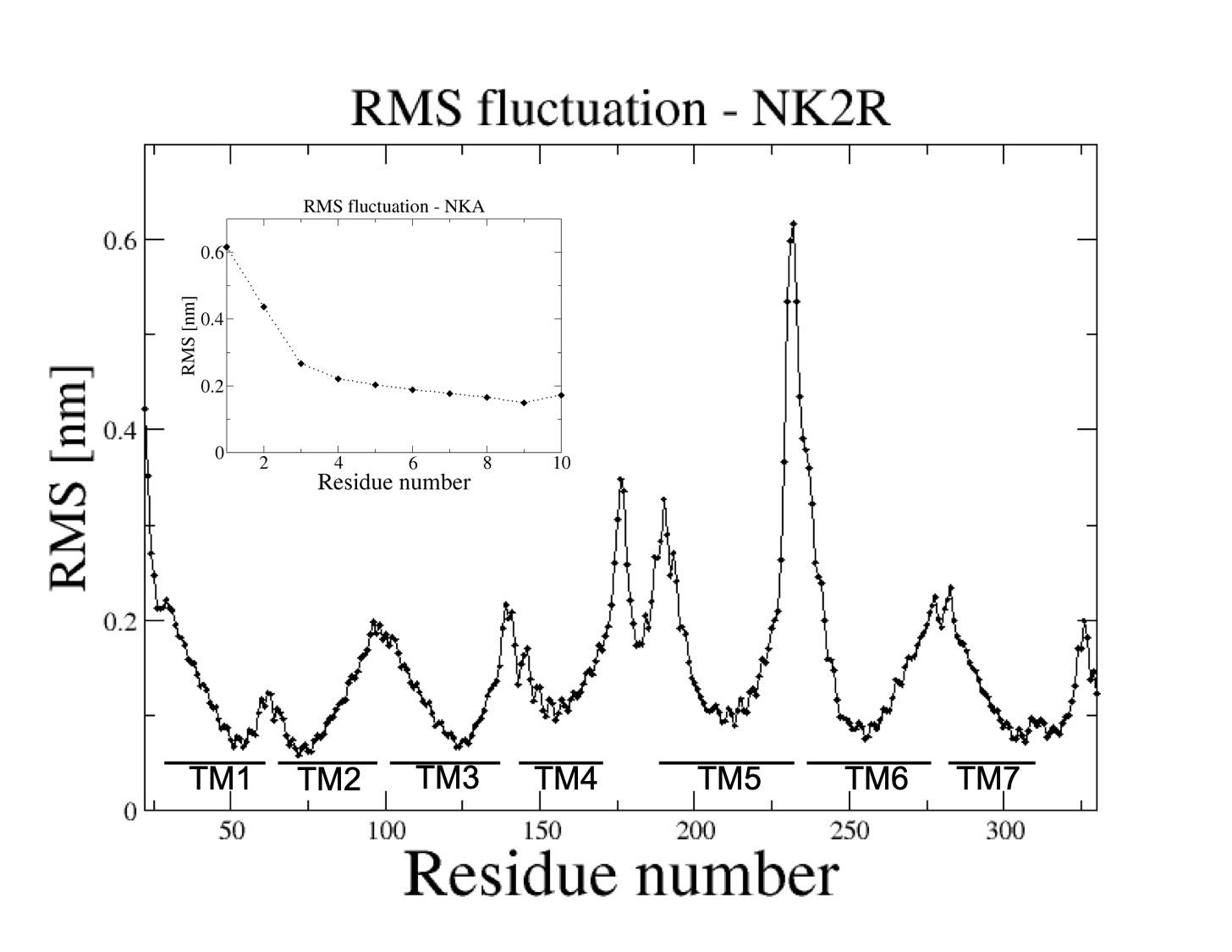


**Figure S2**: RMS fluctuations (for C_α_ atoms) from 2000 ns of MD simulations of NK2R:NKA:Gq complex. The location of the TMs is indicated according to annotation in GPCRdb (37). TM fluctuations are generally low. The insert shows RMS fluctuations of NKA. The N-terminal residues 1-3 of NKA exhibit large fluctuations consistent with their pronounced solvent accessibility and in sharp contrast to the buried C-terminal residues.


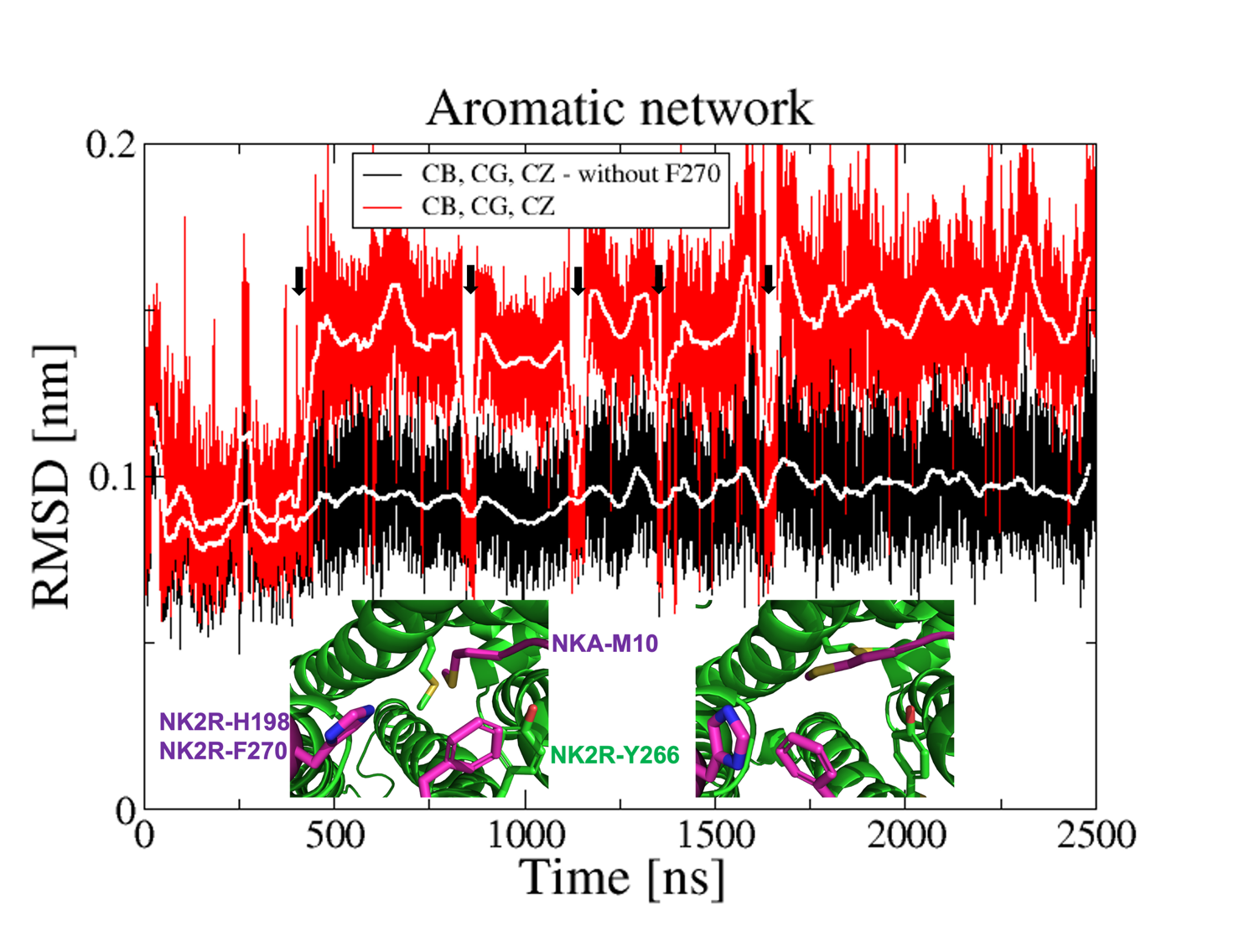


**Figure S3:** Aromatic ring interactions for NK2R-F26, -Y266, -Y269, -F270, -Y280, -Y289, -F293 and NKA-F6 in terms of RMS calculations. Red: only CB, CZ and CB to omit RMS changes caused by ring flips, black: only CB, CZ and CB with F270 excluded. Running averages in white. The sidechain of F270 undergoes conformational change. Black arrows indicate conformational change events. The inserts illustrate the conformation of F270 at the beginning of the trajectory (left insert, T=100ns) and at the end (right insert, T=2400ns).


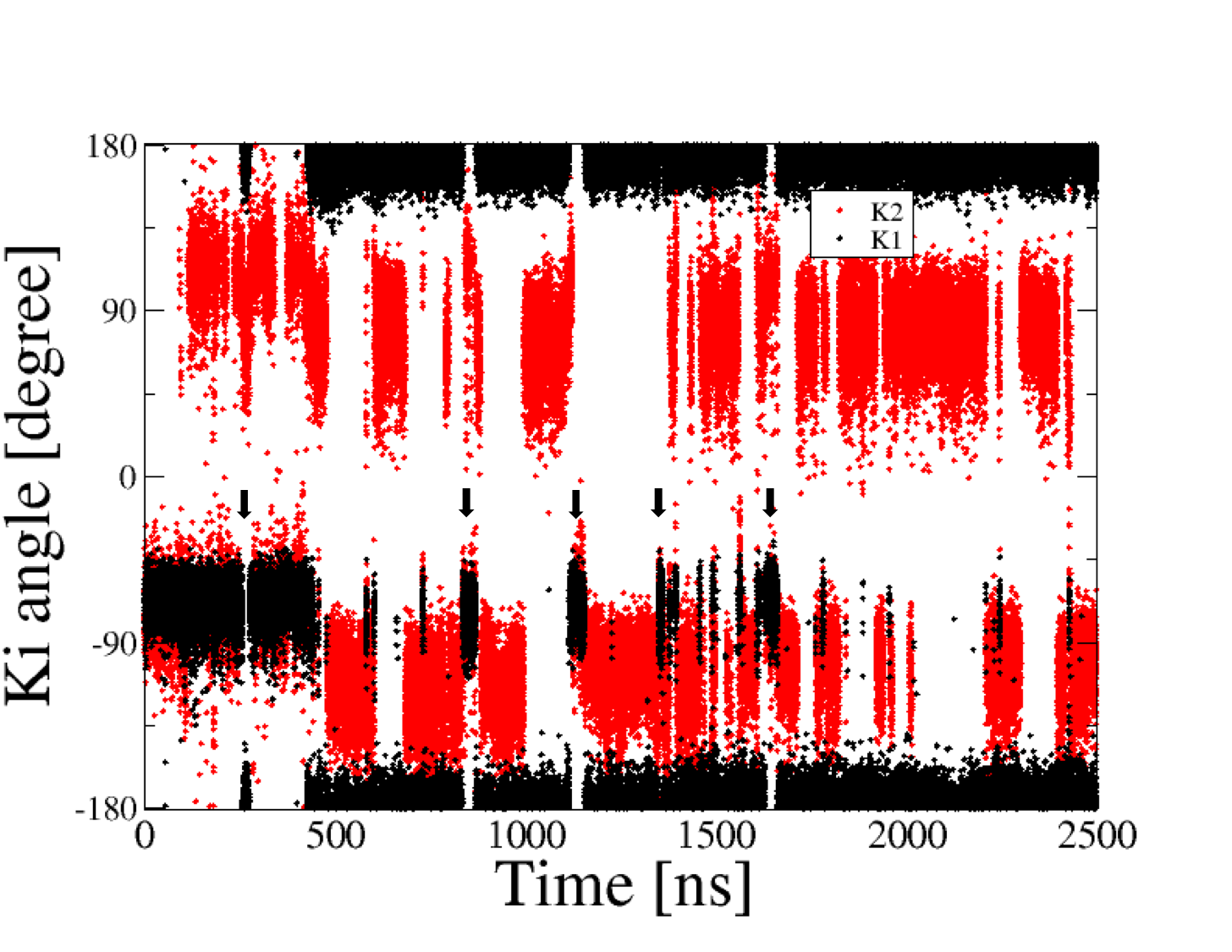


**Figure S4:** κ angles for NK2R-D270, κ1(κ2) in black (red). At the jump at Time=500 the κ1 of NK2R-F270 angle predominantly is in the vicinity of |180| degrees with a few visits to its original conformation as indicated arrows.


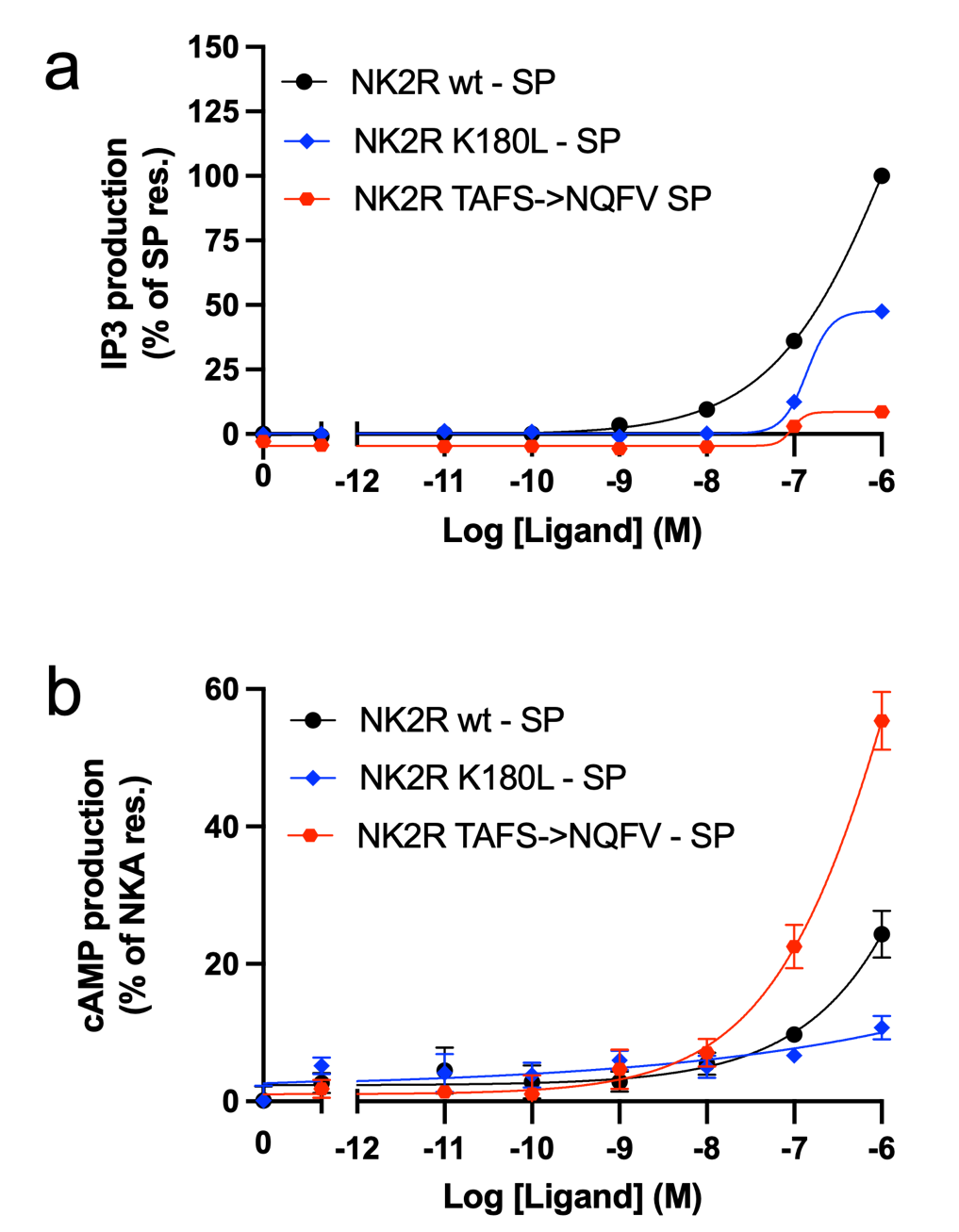


**Figure S5**: SP activation of NK2R, and mutations NK2R-K180L and NK2R-TAFS24-27/NQFV (a) tested in IP_3_ activation and (b) BRET-based cAMP assays. A dramatic impact on activation is observed for NK2R-K180L, consistent with the AF2 model. Interestingly, NK2R-TAFS24-27/NQFV shows enhanced activation in BRET-based assay consistent with it being more NK1R-like. In Table S2 the EC50 and Emax values from functional assays corresponding to panels a and b are tabulated.


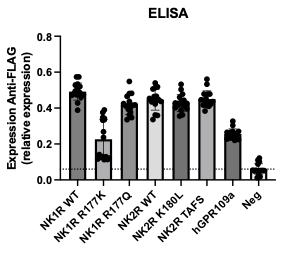


Figure S6: Expression levels for wildtype receptors and mutants. Dotted line represent background in cells transient transfected with empty vector.
