# Supplementary material for: Deciphering specificity and cross-reactivity in tachykinin NK1 and NK2 receptors
Source: J Biol Chem. 2023 Nov 7;299(12):105438. doi: 10.1016/j.jbc.2023.105438 (PMC10724690; doi:10.1016/j.jbc.2023.105438)
Supplement: Table S1 [file mmc2.docx]

| Chimeric construct, ref. (4) | ECL2 | N-terminal | SP [nM] | NKA [nM] |
| --- | --- | --- | --- | --- |
| NK1R1-195, NK2R196-398 | NK1R-R177 | NK1R-NQFS | 0.14 (1) | 12 (13) |
| NK1R1-130, NK2R131-398 | NK2R-K180 | NK1R-NQFS | 5 (36) | 33 (37) |
| NK2R | NK2R-K180 | NK2R-TAFS | 460 (3300) | 0.9 (1) |

**Table S1:** Chimeric exchange of transmembrane segments illustrate interplay between ECL2 and N-terninals. Chimeric constructs, ECL2 and N-terminal specifications, and binding affinities for SP and NKA. Data illustrate the incompatibilities of combining ECL2 and N-terminal segments. The relative binding affinities in parentheses. The important sidechains specified in columns named ECL2 and N-terminal.
