# Supplementary material for: Deciphering specificity and cross-reactivity in tachykinin NK1 and NK2 receptors
Source: J Biol Chem. 2023 Nov 7;299(12):105438. doi: 10.1016/j.jbc.2023.105438 (PMC10724690; doi:10.1016/j.jbc.2023.105438)
Supplement: Table S2 [file mmc3.docx]

|  | Sample ID | Emax | EC50 |
| --- | --- | --- | --- |
| Fig 3 a | Ala1 | 1133 | 7,15e-010 |
|  | Ala2 | 1140 | 6,80e-010 |
|  | Ala3 | 1108 | 5,98e-010 |
|  | Ala4 | 1066 | 6,61e-010 |
|  | Ala5 | 1146 | 2,59e-008 |
|  | Ala6 | NA | NA |
|  | Ala7 | 178 | NA |
|  | Ala8 | 1206 | 8,80e-010 |
|  | Ala9 | 136 | NA |
|  | Ala10 | 898 | 3,19e-008 |
|  | Ref peptide | 1191 | 9,15e-010 |
| Fig 3 c | NK2R WT | 100 | 4,413e-009 |
|  | NK2R K180L | 18,58 | 1,063e-007 |
|  | NK2R TAFS->NQFV | 3,63 | 1,889e-008 |
| Fig 3 d | NK2R WT | 85,27 | 6,299e-009 |
|  | NK2R K180L | 5,04 | 2,467e-010 |
|  | NK2R TAFS->NQFV | 12,57 | 2,989e-008 |
| Fig 3 e | NK2R WT | 99,98 | 5,212e-009 |
|  | NK2R K180L | 18,67 | 6,953e3 |
|  | NK2R TAFS->NQFV | 35,83 | NA |
| Fig 5 a | NK1R WT NKA | 100 | 3,626e-009 |
|  | NK1R WT SP | 96,75 | 2,520e-009 |
|  | NK2R NKA | 103,8 | 4,413e-009 |
|  | NK2R SP | 45,21 | 3,329e-008v |
| Fig 5 b | NK1R WT NKA | 100 | 2,590e-008 |
|  | NK1R WT SP | 110,21 | 4,179e-010 |
|  | NK2R NKA | 99,98 | 5,212e-009 |
|  | NK2R SP | 24,32 | 2,259e-002 |
| Fig 7 a | NK1R WT | 100 | 2,520e-009 |
|  | NK1R R177K | NA | NA |
|  | NK1R R177Q | 121,39 | 1,844e-008 |
| Fig 7 b | NK1R WT | 110,21 | 4,179e-010 |
|  | NK1R R177K | 28,90 | 7,827e-008 |
|  | NK1R R177Q | 80,79 | 1,016e-007 |
| Fig 7 c | NK1R WT | 100 | 3,626e-009 |
|  | NK1R R177K | NA | NA |
|  | NK1R R177Q | 59,29 | 7,025e-006 |
| Fig 7 d | NK1R WT | 108,7 | 2,590e-008 |
|  | NK1R R177K | 10,7 | 1,076e3 |
|  | NK1R R177Q | 13,1 | 7,776e2 |
| Fig s6 a | NK2R WT | 100 | 1,950e-006 |
|  | NK2R K180L | 47,59 | 1,396e-007 |
|  | NK2R TAFS->NQFV | 8,56 | 9,443e-008 |
| Fig s6 b | NK2R WT | 24,32 | 2,259e-002 |
|  | NK2R K180L | 10,73 | NA |
|  | NK2R TAFS->NQFV | 55,36 | 3,444e-006 |

**Table S2:** Table with EMAX and EC50 values for non-linear fitted dose-response curves.
